# Determinants and processes of HIV status disclosure to HIV - infected children aged 4 to 17 years receiving HIV care services at Baylor College of Medicine Children’s Foundation Tanzania, Centre of Excellence (COE) in Mbeya: a cross-sectional study

**DOI:** 10.1186/s12887-015-0399-3

**Published:** 2015-07-15

**Authors:** Mary S. Nzota, Joseph KB. Matovu, Heather R. Draper, Rose Kisa, Suzanne N. Kiwanuka

**Affiliations:** Mbeya Consultant Hospital, P.O. Box 419, Mbeya, Tanzania; School of Public Health Makerere University College of Health Sciences, P.O. Box 7072, Kampala, Uganda; Baylor College of Medicine International Pediatric AIDS Initiative at Texas Children’s Hospital, Houston, TX 77030 USA

**Keywords:** Disclosure, HIV- infected children, Determinants, Processes

## Abstract

**Background:**

Disclosure of HIV sero-status to HIV-infected children is associated with reduced risk of death and better adherence to antiretroviral drugs. However, caregivers find it difficult to determine when and how they should disclose the HIV sero-positive status to HIV-infected children. In this study, we assessed the determinants and processes of HIV status disclosure to HIV-infected children aged 4 to 17 years receiving HIV care services at the Baylor College of Medicine Children's Foundation Tanzania, Centre of Excellence (COE) in Mbeya.

**Methods:**

This was a cross-sectional study conducted among 334 caregivers of HIV positive children attending the Baylor COE in Mbeya, Tanzania. Data were collected using quantitative and qualitative research methods. Quantitative data were collected on socio-demographic characteristics of children and caregivers using an interviewer-administered questionnaire. Data were entered into Epi-Info version 3.5.1 and analyzed using STATA version10. Univariable and multivariable logistic regression analyses were conducted to obtain odds ratios (OR) and 95 % confidence intervals (95 % CI) associated with disclosing HIV positive status to HIV-infected children. Qualitative data were collected on the processes used in accomplishing the HIV status disclosure event using case histories and key informant interviews and analyzed manually using latent analysis techniques.

**Results:**

About one-third of the caregivers (32.6 %) disclosed the children’s HIV sero-positive status to them. Disclosure was more likely among children 10 years or older (adjusted OR [AOR] = 8.8; 95 % CI: 4.7, 16.5), caregivers with knowledge about HIV disclosure (AOR = 5.7; 95 % CI: 2.3, 13.7) and those earning more than Tsh 99,999 (US $62.5) per month (AOR = 2.4; 95 % CI: 1.3, 4.5). Qualitative findings showed that caregivers used a diversity of approaches to complete the HIV status disclosure event including direct, third-party, event-driven and use of drawings.

**Conclusions:**

Our study shows that disclosure is common among older children and is largely driven by the caregivers’ knowledge about HIV status disclosure and monthly earnings. HIV status disclosure was accomplished through a variety of approaches. These findings suggest a need to provide caregivers with knowledge about HIV status disclosure approaches to improve HIV status disclosure to HIV-infected children.

**Electronic supplementary material:**

The online version of this article (doi:10.1186/s12887-015-0399-3) contains supplementary material, which is available to authorized users.

## Background

Despite a 43 % decline in Human Immunodeficiency Virus Infection (HIV) among children in 21 priority countries in Africa, 87.5 % of new pediatric infections in 2013 were in Sub-Saharan Africa [23]. Evidence from a recent systematic review shows that the majority of the children less than 18 years living with HIV in resource-limited settings, including sub-Saharan Africa, are not aware of their own HIV status [24]. This has implications for their enrolment into HIV care as well as adherence on antiretroviral treatment [18]. Although the benefits associated with HIV status disclosure are known, the proportion of children who know their HIV status varies, ranging between 0-69 % [18]. Disclosing to HIV-infected children about their HIV sero-positive status (i.e. informing HIV-infected children that they have HIV) has been associated with proper adherence to medications and can help children to cope with the stresses associated with HIV infection [6, 7, 9, 14, 16]. However, studies suggest that HIV status disclosure is often delayed until after 10 years of age because it is believed that prior to this age children will not be able to understand the information or deal with the stigma associated with the diagnosis [18]. This has implications for HIV status disclosure to young children who are considered to be less able to understand the meaning of HIV infection [1].

Tanzania’s National HIV and AIDS Control Program recommends partial HIV status disclosure to HIV-infected children at four years with full disclosure completed by eight to ten years [22]. At Baylor Centre of Excellence (COE), disclosure depends on the cognitive maturity of a child, but it begins at the age of four years and full disclosure is expected to have been completed at the age of 12 years. Reports show that only 24 % of HIV- infected children in the facility were completely aware of their HIV status in 2011 [2]. The reasons for this low level of HIV status disclosure are not well documented. In addition, there is limited information about the different approaches used to accomplish the disclosure process to HIV-infected children, yet it is imperative for such children to know their own HIV sero-positivity.

Studies suggest that HIV disclosure can be accomplished through direct, third party and event-driven approaches. Direct approaches, which involve telling the target directly (face to face) that one has got HIV, have been described in several studies [10, 11, 15]. Third party approaches involve entrusting a third party (such as the child’s health care provider) to inform the target about their HIV infection status [15]. The event-driven approaches involve placing HIV-related medications and referral forms where they can be seen to stimulate the target’s curiosity and initiate disclosure [10]. In most cases, these approaches have been utilized to accomplish HIV status disclosure among adults who are afraid of their partners’ reactions or are less confident about their communication skills [10] but not to facilitate HIV status disclosure to HIV-infected children. This creates a missed opportunity for informing HIV-infected children about their HIV-positive status. To bridge this gap, we conducted a study to assess the determinants and processes of HIV status disclosure to children aged 4 to 17 years, receiving HIV care services at the Baylor COE in order to improve the current disclosure practices.

## Methods

### Study site

The study was conducted at the Baylor COE, situated within the Mbeya Consultant Hospital in the Southern highland zone of Tanzania. The COE has been operating since 2009 under the Walter Reed Program until early 2011 when it started operating as an independent entity caring for children. Since March 2012, the Baylor COE has provided a wide range of services including provision of antiretroviral drugs (ARVs), HIV counseling and testing and disclosure to approximately 1,500 children living with HIV in Mbeya region and conducts outreach programs in the Southern highland zone of Tanzania. The Baylor COE uses a “flipbook” designed by Ms. Feinglass (a volunteer public health specialist) following focus groups with Botswana healthcare providers and parents to guide HIV status disclosure to HIV infected children. The visual aids were translated by Baylor COE team in Mbeya to guide the health care providers and caregivers.

### Study design and population

This was a cross-sectional study that employed quantitative and qualitative data collection methods. Data collection was done between March and April 2012. Quantitative data collection methods involved the use of structured questionnaires that were administered to caregivers of HIV-infected children receiving HIV care at the Baylor COE by trained research assistants. Qualitative data collection methods included case histories with purposely selected caregivers and key informant interviews with health care workers working at the Baylor COE.

### Sample size determination

Using the Kish-Leslie formula, with a 5 % level of precision, a standard critical value of 1.96 (representing 95 % confidence) and a proportion of caregivers that had ever disclosed the HIV-positive status to HIV-infected children of 0.29 [25], we obtained a sample size of 317. After accounting for an estimated 5 % non-response, a sample of 334 caregivers was obtained.

### Data collection procedures

Quantitative data were collected using pre-tested, semi-structured interviewer-administered questionnaires (Additional file 1). All study documents were translated to and from Swahili and pre-tested to enhance consistency and validity. The questionnaires were administered to eligible caregivers of children aged 4 to 17 years whose children were receiving HIV care services at the Baylor COE during the data collection period. On each clinic day, a computer-generated random sample of HIV-infected children already enrolled at the Baylor COE was obtained and their caregivers were invited to participate in the interview. Participants were recruited consecutively on subsequent clinic days until the required sample size was obtained. Consenting caregivers were interviewed privately and in the absence of their children to maintain confidentiality.

Qualitative data were collected using a key informant interview guide and the HIV status disclosure processes were captured using case history narratives augmented with illustrative diagrams or drawings. Data were collected on the different processes used by caregivers (who had ever disclosed) to disclose HIV positive status to HIV- infected children. Key informant interviews (KIIs) were conducted with six purposely selected health care workers (i.e. 4 counselors and 2 doctors) working at the Baylor COE. Since these health workers were trained on HIV status disclosure to HIV- infected children and since they interacted more often with caregivers, they were considered to be knowledgeable about the HIV status disclosure events and were interviewed as key informants. Key informant interviewees were asked about how they completed the disclosure event, including whether or not they made reference to the “flipbook” when deciding how to disclose to HIV-positive children their HIV sero-positive status. Case histories were conducted with ten purposely selected caregivers to document the processes and timelines for HIV status disclosure. In a typical case history, caregivers were asked to explain the key events leading to the HIV status disclosure event, from the time they first came into contact with the HIV-positive child to the time they told them that they were HIV-positive. In all interviews, probes were used to gain further insights on how the actual HIV status disclosure event was actually accomplished including the time it took the caregiver to complete this process. The case histories and KIIs interviews were tape-recorded and transcribed verbatim. Interviews took between 30 to 45 minutes.

### Measurement variables

The outcome variable was HIV status disclosure to HIV-infected children, measured dichotomously with the question “*Have you disclosed to your child his/her HIV-positive status?*” Another question, “*What exactly did you tell the child about his/her illness?*” was used to assess whether the child has been told that she/he has HIV infection. Those caregivers who answered “Yes” to the first question and directly mentioned to the child that he/she had HIV /AIDS for the second question were regarded as caregivers who had disclosed to the child. Those who responded to the first question in the negative; i.e. those who said “No”, were not asked the second question, and were automatically considered to not have disclosed to the child. Predictor variables describing demographic characteristics of the children were age in years and education, while variables describing socio-demographic characteristics of the caregivers were sex, age, education and knowledge about HIV status disclosure (Caregivers were asked about whether or not they have heard about or actually been involved in disclosure of HIV sero-positive status to a child living with HIV, and those who responded in the affirmative way were considered to be knowledgeable about HIV status disclosure). Other variables included the caregiver’s occupation, relationship with the child and family income level.

### Data quality control

Three research assistants were trained for five days and equipped with interviewing and probing skills. Data collection was supervised by the Principal Investigator and the research team met every day to ensure that the collected data were accurate. Data were cleaned, checked for any inconsistencies and coded by the Principal Investigator (MN) before data analysis. Data were entered into Epi-Info version 3.5.1. About 5 % of the questionnaires were double-data entered to check for accuracy of data entries made. All entered data were kept secure by the Principal Investigator, on a password-protected computer with limited access to the research team.

### Data analysis

Quantitative data analysis was done using STATA statistical software version 10.0. Descriptive statistics were computed to summarize data and obtain frequencies. Comparisons between the predictor variables such as sex, age, education and relation of the caregivers to the child and HIV status disclosure were done using the Chi-square test. Univariable logistic regression was conducted to identify predictor variables that were associated with disclosing HIV sero-positive status to HIV-infected children. Any variable with a p-value <0.10 in the univariable analysis was included in the multivariable logistic regression model using a forward stepwise approach. Empirically known potential confounders from scientific literature (such as caregiver’s age and sex) were included in the multivariable model, even if they had a p-value >0.10. For the multivariable analysis, p-values less than 0.05 (p < 0.05) were considered to be statistically significant.

Qualitative data were analyzed thematically, following content analysis techniques. Copies of transcribed data were printed and read by authors to identify quotes pertaining to the different HIV status disclosure processes (direct, third-party, event-driven and the use of the drawings). The principal investigator (MN) identified and coded the findings by theme using an Excel worksheet and shared them with co-authors via email. These co-authors reviewed the merged quotes to determine the extent to which they portrayed the different HIV status disclosure processes, and aligned with identified themes in the data, using Google Docs. Quotes that seemed similar were grouped together and evaluated further for their “richness” in textual data. Quotes that were considered to have richer textual data than others were considered for use in reporting the findings. MN copied and pasted (by theme) all selected quotes into a MS Word document file and edited them to improve their clarity. All selected quotes are presented verbatim in the results.

### Ethical considerations

The study obtained ethical approval from Makerere University School of Public Health Higher Degrees Research and Ethics Committee, Mbeya Consultant Hospital Institutional Review Board (IRB) and by the IRB at Baylor College of Medicine, Houston, Texas, USA. Written informed consent was also obtained from caregivers (aged 18+ years) of HIV infected children receiving HIV care services at the Baylor COE.

## Results

### Quantitative findings

#### Characteristics of the participants

The study consisted of 334 caregivers of which 273 (81.7 %) were female and 166 (49.7 %) were biological mothers. 139 (41.6 %) were aged between 31 to 40 years with a mean age of 40 years (SD ± 11.6). 203 (61 %) of the caregivers had primary education, and 134 (40.1 %) earned a monthly income of more than Tsh 99,999 (US $62.5). The mean age of the children was 9.4 years (SD ±3.5), with slightly more than half of the children (170/334) aged 10 years and above. Seventy two per cent of the children reported that they had at least primary education (240/334) (Table 1).

**Table 1:** Socio-demographic characteristics of caregivers and children

Variable	Frequency N = 334	Percentage (%)
Caregivers’ characteristics		
Sex		
Male	61	18.3
Female	273	81.7
Age		
<30	68	20.4
31-40	139	41.6
41-50	64	19.2
51-60	38€	11.4
60+	25	7.5
Education		
None	38	11.4
Primary	203	60.8
Post primary	93	27.8
Occupation		
Farmer	100	29.9
Employed	63	18.9
Not Employed	171	51.2
Income (Tsh)		
<=49,999	126	37.7
50,000-99,999	74	22.2
>99,999	134	40.1
Relationship to the child		
Biological mother	166	49.7
Biological father	40	12.0
Other relationship^a^	128	38.3
Knowledge of HIV disclosure		
Don’t have	92	24.5
Have Knowledge	242	72.5
Children’s Characteristics		
Sex		
Male	156	46.7
Female	178	53.3
Age of the child		
4-9 years	164	49.1
10-17 years	170	50.9
Education of the child		
Preprimary	94	28.1
Primary or higher	240	71.9

^a^Other relationship includes brothers, sisters, uncles, aunties, or grandparents. Tsh, Tanzania Shillings

#### Determinants of HIV status disclosure

Overall, 32.6 % of caregivers disclosed the HIV positive status to the HIV- infected children (Table 2). In univariable logistic regression analysis, HIV status disclosure was 2.7 times higher among caregivers who reported a monthly income of more than Tsh 99,999 (US $62.5) compared to those who reported less than Tsh 49,999 (US $ 31.2) per month (OR = 2.7; 95 % CI: 1.6, 4.7; p < 0.001). Caregivers who had knowledge about HIV status disclosure were 8.8 times more likely to disclose to HIV- infected children (Odds Ratio [OR] = 8.8; 95 % CI: 3.9, 19.9; p < 0.001) than those who were not knowledgeable. Older age of the caregiver (e.g. 41 to 50 years; OR = 6.0; 95 % CI 2.7, 13.3; p < 0.001) and older age of the children (e.g. children 10 years or older; OR = 11.2; 95 % CI 6.1, 20.3; p < 0.001) were significantly associated with HIV status disclosure.

**Table 2:** Univariable and multivariable logistic regression analysis of the determinants of HIV status disclosure to HIV infected children attending the Baylor COE in Mbeya, Tanzania, 2012

Predictor variables	Not disclosed	Disclosed	UAOR (95 % CI)	p-value	AOR (95%CI)	p-value
Overall	225(67.4 %)	109(32.6)				
Caregivers’ variables						
Sex						
Male	35(15.6)	26(23.8)	1.0(ref)		1.0(ref)	
Female	190 (84.4)	83(76.2)	0.6(0.3-1.0)	0.070	0.7(0.3-1.2)	0.768
Age						
<30	56(24.9)	12(11.0)	1.0 (ref)		1.0(ref)	
31-40	101(44.9)	38(34.9)	1.7(0.8-3.6)		1.2(0.6-2.9)	
41-50	28(12.4)	36(33.0)	6(2.7-13.3)***		3.2(1.3-8.0)	
51-60	24(10.7)	14(12.8)	2.7(1.1-6.7)*		1.4(0.5-4.0)	
60+	16(7.1)	9(8.3)	2.6(0.9-7.3)	0.000	1.6(0.5-5.3)	0.410
Education level						
None	27(12.0)	11(10.1)	1.0(ref)		1.0(ref)	
Primary education	145(64.4)	58(53.2)	0.9(0.4-2.1)		1.3(0.6-3.1)	
Post primary education	53(23.6)	40(36.7)	1.8(0.8-4.2)	0.046	2.0(0.8-5.1)	0.430
Occupation						
Farmer	65(28.9)	35(32.1)	1.0(ref)		1.0(ref)	
Employed	31(13.8)	32(29.4)	1.9(1.0-3.6)*		1.2(0.5-2.7)	
Not employed	129(57.3)	42(38.5)	0.6(0.3-1.0)**	0.001	0.6(0.3-1.1)	0.122
Income(Tsh)						
<=49,999	98(43.6)	28(25.7)	1.0(ref)		1.0(ref)	
50,000-99,999	52(23.1)	22(20.2)	1.5(0.8-2.8)		1.3(0.6-2.7)	
>99,999	75(33.3)	59(54.1)	2.7(1.6-4.7)***	0.001	2.4(1.3-4.5)*	0.006
Relation to the child						
Biological mother	123(54.7)	43(39.4)	1.0(ref)		1.0(ref)	
Biological father	25(11.1)	15(13.8)	1.7(0.8-3.5)		1.4(0.6-3.4)	
Others	77(34.2)	51(46.8)	1.9(1.1-3.1)**	0.032	1.5(0.8-2.6)	0.232
Knowledge of HIV disclosure						
Don’t have knowledge	85(37.8)	7(6.4)	1.0(ref)		1.0(ref)	
Have knowledge	140(62.2)	102(93.6)	8.8(3.9-19.9)***	0.000	5.7(2.3-13.7)***	0.000
Children’s variables						
Sex						
Male	107(47.6)	49(44.9)	1.0(ref)		1.0(ref)	
Female	118(52.4)	60(55.1)	1.1(0.7-1.7)	0.655	0.6(0.3-1.2)	0.196
Age						
4-9 years	148(65.8)	16(14.7)	1.0(ref)		1.0(ref)	
10-17 years	77(34.2)	93(85.3)	11.2(6.1-20.3)***	0.000	8.8(4.7-16.5)***	0.000
Education						
Preprimary	88(39.1)	6(5.5)	1.0(ref)		1.0(ref)	
Primary or higher	137(60.9)	103(94.5)	11.0(4.6-26.2)***	0.000	2.4(0.8-6.7)	0.088

UAOR-Unadjusted odds ratio, AOR-Adjusted odds ratio, CI-Confidence interval

*Statistically significant at p < 0.05, **p < 0.01, *** p < 0.001

After adjusting for potential confounders in the multivariable analysis, the factors that remained significantly associated with HIV status disclosure to HIV- infected children were: caregiver’s monthly income of more than Tsh 99,999 (US $62.5) (adjusted OR (AOR) = 2.4; 95 % CI: 1.3,4.5; p = 0.02), knowledge about HIV status disclosure (AOR = 5.7; 95 % CI 2.3,13.7; p < 0.001) and children 10 years or older (AOR = 8.8; 95 % CI 4.7,16.5; p < 0.001). An interaction between knowledge and income made HIV status disclosure 5.1 times higher among caregivers who reported a monthly income of more than Tsh 99,999(US $62.5) compared to those who reported less than Tsh 49,999 (US $ 31.2) per month (OR = 5.1; 95 % CI: 1.1, 24.2; p = 0.05). When we included an interaction term in the model, the effect of knowledge on HIV disclosure to HIV infected children reduced by 40 % and this effect remained significant at 5 %.

### Qualitative findings

#### HIV status disclosure processes

We identified a number of HIV status disclosure approaches ranging from use of direct, third-party, and event-driven approaches as well as use of drawings of a bad person and a policeman to accomplish the HIV disclosure event. Caregivers and health care workers used different approaches to accomplish the HIV status disclosure event to HIV- infected children. Caregivers disclosed mainly through third-party disclosure although direct and event-driven approaches were also used. On the other hand, health workers accomplished the disclosure event mainly through the use of the drawings. Details about these approaches are presented below. For purposes of maintaining anonymity and protect identities, children’s real names were replaced with pseudonyms.

#### Event-driven approach

Some caregivers reported that they did not have the courage and skills to disclose the HIV sero-positive status of their children to them. This necessitated them to use events or occasions where HIV infection was mentioned as a way of initiating the conversations which led to the disclosure of HIV status to HIV positive children as reported by Jacob’s uncle;

*“Jacob likes watching movies, one day I had jokes with him when watching the movie about a lady who got an accident and during that accident she got HIV. The lady had never had sexual intercourse before … I asked him if he knows why he is taking medication. I told him that he is HIV positive and he got the disease from his parents. The movie encouraged me to tell him he was infected with HIV because I was afraid to tell him the truth before” (reported by Jacob’s uncle).*

#### Third-party approach

Some caregivers reported that they were unable to disclose HIV sero-positive status to the children even when the child was tired of taking ARVs. They requested for assistance from the health-care worker to disclose to the child, hoping that professional support would help them deal with any questions the children might ask. Rebecca’s case presents a vivid description of a third-party disclosure event;

*“My younger sister asked me why she was the only person taking medications every day… I did not tell her anything until she reached 10 years when she asked again and insisted on knowing the truth. My other sister and I requested the nurse to help us. The nurse told her; “you have HIV like me … I am also HIV positive and using medications”. Rebecca told the nurse, “you are lying! Why are you so fat?” the nurse replied, “if you take the medications you will be healthy like me*”.

Using a third-party to complete the HIV status disclosure event tended to take longer than other approaches. For instance, it took two years for Rebecca to know her HIV positive status from the time she inquired about her health status.

#### Direct approach

In order to ensure that children adhere to ARV medications, some caregivers told their children directly that they had HIV because they thought that if the child knew the truth, he/she would not refuse the medications. However, this was normally the case with older (aged nine years or more) children. Edward’s father told us that he told Edward about his HIV infection without mincing words:

*“[I called him and said…] Edward, you will be taking these medications for the rest of your life, because you are suffering from AIDS” (reported by Edward’s father).*

The disclosure happened when Edward was 10 years old, and he was devastated but the father calmed him down and told him to accept the situation.

#### Use of illustrative diagrams or drawings

When asked if they have ever disclosed HIV-positive status to HIV- infected children, and if so, how they did it, almost all key informants (i.e. health-care providers) reported using drawings of a bad person (e.g. a thief) and a policeman during disclosure to children aged 6 up to about or even more than 12 years. This was achieved through the use of visual aids adapted and translated from the flipbook. For children aged more than 11 years, health care providers assessed their knowledge of HIV/AIDS issues before disclosing to them.

*“We relate HIV to a bad person and the CD4 count to a policeman. When children understand about them, we disclose to them that they have HIV which is a bad person and the policeman is the CD4 cell which fights with the bad person. The CD4 will be able to fight the bad person if they take medications” (reported by pediatric counselor 2).*

## Discussion

This study of HIV status disclosure to HIV- infected children aged 4 to 17 years attending the Baylor COE in Mbeya urban, Tanzania, found that the prevalence of HIV status disclosure by caregivers to HIV positive children receiving HIV care services at the Baylor COE was 32.6 %. This low level of disclosure could be explained by the fact that most caregivers prefer to delay disclosure up to when a child has reached nine years or older since they believe that, older children have cognitive maturity and are able to understand the importance of taking ARVs [4, 5]. Indeed, our findings show that older age of the child was a significant determinant for disclosure of HIV-positive status to HIV- infected children. However, since 51 % of the children in HIV care were aged 10 or more years, we expected slightly higher levels of HIV status disclosure to HIV-infected children but this was not the case. Most likely, caregivers were not sure how to disclose the HIV positive status to HIV- infected children or did not know that disclosing the HIV-positive status to HIV-infected children was necessary. Consequently, many HIV-positive children including those older than nine years remain unaware of their HIV positive status and this has implications on their ability to adhere to HIV treatment [13, 19, 25]. The low levels of disclosure to HIV positive children have been reported in other studies. In Zambia, for instance, Menon et al. [12] found that only 37.8 % of children aged 8 to 17 years knew their HIV status while a much lower proportion was observed in a cross-sectional study done in Ethiopia [10] where only 17.4 % of children aged 6 to 14 years knew their HIV status. These findings suggest a need for interventions aimed at improving HIV status disclosure to HIV-infected children through different approaches including those documented in this study.

We found that caregivers’ monthly income and their knowledge about HIV status disclosure were positively and significantly associated with HIV status disclosure to HIV- infected children. The finding that caregivers with high monthly income disclosed more to HIV- infected children than those who had low income has also been reported in a systematic review by Weiner et al. [5]. This implies that poverty may be a barrier to HIV status disclosure possibly because the caregiver has less access to resources including psychosocial support [5, 20]. However, this contradicts findings from some studies which report that caregivers who have financial problems disclose early [3, 8]. The association between knowledge about HIV status disclosure and actual disclosure of HIV positive status to HIV- infected children could possibly be due to the counseling that the caregivers receive when they bring children for routine care at the facility. These findings suggest the need for sensitizing and educating caregivers about HIV status disclosure in order to increase the proportion of children who know their HIV status [21].

In terms of HIV disclosure processes, the most common approaches caregivers used to accomplish the disclosure process was mainly through third-party disclosure and through the use of drawings, although direct and event-driven approaches were also used. To the best of our knowledge, the approaches used to disclose to HIV-infected children reported in this study, are less documented compared to approaches used to accomplish HIV status disclosure among adults [11, 17]. The use of a third party (i.e. another person(s) to do the disclosure) might imply that caregivers may not be well equipped to disclose to HIV- infected children. To improve HIV status disclosure, health care providers should educate caregivers about the importance of HIV status disclosure and equip them with necessary skills and materials to enable them to perform disclosure to HIV- infected children with simple examples. As noted, health care providers reported using drawings of a bad person and a policeman to facilitate the disclosure process. More studies are needed to investigate the suitability of using illustrative pictures such as those used by the health workers in this study.

This study had several limitations. Some groups, such as caregivers without knowledge of HIV disclosure, were not well represented. As the study was conducted among caregivers in a clinical setting, the disclosure rate may have been overestimated. However, it was not feasible for practical and ethical reasons to interview caregivers not seeking services at the facility. The cross-sectional nature of the study precluded assessment of HIV status disclosure dynamics over time. In addition, the study findings were based on respondents' self-reports which might have biased reporting, although this was likely minimized by the use of probes and check questions to enhance study rigor. The existence of the flipbook might have also caused disclosure practices to differ from usual practice. Lastly, we did not assess the effect of ARV treatment of the child, caregiver HIV status, and relationship of the caregiver to the child on disclosure. A relative strength was the use of mixed qualitative and quantitative methods which provided information that could be instrumental in designing strategies to promote early HIV status disclosure to HIV- infected children.

Nevertheless, despite these limitations, our study has clear public health implications. The fact that caregivers with knowledge of HIV status disclosure were more likely to disclose the HIV-positive status to HIV- infected children necessitates the need to provide caregivers with knowledge and disclosure skills, and to develop guidelines for sensitizing health-care providers on the importance of disclosing to HIV positive children about their HIV sero-positive status. There is also a need for standardizing the mechanisms that health workers use to complete the HIV status disclosure process to avoid a situation where different health workers use different approaches that cannot be compared or measured for efficacy. On the other hand, health care workers and caregivers should work together to determine the best disclosure processes that are suitable to each child’s circumstances.

## Conclusion

This study demonstrates that older age of the children, higher caregiver monthly income and knowledge about HIV status disclosure were strong determinants of HIV status disclosure and that the HIV disclosure process was mainly accomplished through third-parties as well as drawings, although direct and event-driven approaches were also used by caregivers. Considerations should be given to revising national HIV disclosure guidelines to include improving caregiver’s knowledge on the importance of disclosing to HIV- infected children. Low income families should be targeted for additional support on HIV status disclosure to HIV-infected children. Based on this study, many approaches can be used by caregivers and health care workers in disclosing HIV status to HIV- infected children. The effectiveness of these approaches on the children’s understanding of their HIV status and the ultimate effect of these approaches on ART adherence are of particular interest and may warrant further studies.

## Additional file

Additional file 1:
**Study Questionnaire.**

## Abbreviations

AIDS: Acquired Immune Deficiency Syndrome
AOR: Adjusted Odds Ratio
COE: Centre of Excellence
HIV: Human Immunodeficiency Virus
IRB: Institution Review Board
OR: Odds Ratio
TACAIDS: Tanzania Commission for Acquired Immune Deficiency Syndrome
TSH: Tanzanian Shillings
UNAIDS: Joint United Nations Program on HIV/AIDS
US: United States
