# Supplementary material for: Determinants and processes of HIV status disclosure to HIV - infected children aged 4 to 17 years receiving HIV care services at Baylor College of Medicine Children’s Foundation Tanzania, Centre of Excellence (COE) in Mbeya: a cross-sectional study
Source: BMC Pediatr. 2015 Jul 15;15:81. doi: 10.1186/s12887-015-0399-3 (PMC4502565; doi:10.1186/s12887-015-0399-3)
Supplement: Additional file 1: — Study Questionnaire. [file 12887_2015_399_MOESM1_ESM.pdf]

STUDY QUESTIONNAIRE; DETERMINANTS AND PROCESSES OF HIV STATUS DISCLOSURE TO HIV - INFECTED CHILDREN AGED 4 TO 17 YEARS RECEIVING HIV CARE SERVICES AT BAYLOR COLLEGE OF MEDICINE CHILDREN'S FOUNDATION TANZANIA, CENTRE OF EXCELLENCE (COE) IN MBEYA: A CROSS-SECTIONAL STUDY

Section A: General Information

Name of research assistant………………………………… Questionnaire number------- ---------

Country…………………….. District …………………………….. Place of interview-----------

Date……dd…/…mm………/yyyy………..

Time of start of interview___/ Hr: __ _/___ Min. Time of finish_____/Hr___/___min

Section B: <u>Socio Demographic Characteristics & Background Information of the caregivers</u>

1. Date of birth …dd……/…mm………/……yyyy…..

2. Age of the caregiver in completed years-----------

3. Name of the village where the caregiver is coming from -----------------

4. Respondent sex

   1. Male 2.Female

5. What is your marital status?

   1. Never married
   2. Married
   3. Cohabiting
   4. Divorced/separated
   5. Widowed
   6. Others (Specify)_____________

6. What is your religious affiliation?

   1. Catholic
   2. Protestant
   3. Muslim
   4. Others (Specify)_____________________

7. Have you ever attended school?

1. Yes
2. No

8. If yes what was the highest education level attained?

1. Primary education
2. Secondary education
3. Advanced secondary education
4. Certificate
5. University level and above

9. What is your occupation?

1. Not employed
2. Subsistence farmer
3. Business person
4. Civil servant
5. Others (Specify) _________

10. What is your tribe?

1. Kyusa
2. Safwa
3. Ndali
4. Malila
5. Kinga
6. Others (specify)______________________

11. Do you have any income per month?

1. Yes
2. No

12. How much money do you earn per month?__________

Socio Demographic Characteristics & Background Information of the child

13. What relationship do you have with the child?

1. Biological mother
2. Biological father
3. Grandmother
4. Grandfather
5. Other (specify)__________________

14. Who is the child living with? --------------------------------

15. What is your child's date of birth? …dd…/………mm…/…yyyy……..

16. Age of a child in complete year's ______

17. Sex of a child ______

1. Male 2. Female

18. What is Education level of your child?

1. Not in school
2. Kindergarten
3. Primary level
4. Secondary level
5. Others (Specify)_____________________

Proportion of caregivers who have disclosed HIV status to HIV infected children

19. Have you heard about disclosure of HIV sero-positive to a child living with HIV?

1. Yes
2. No

20. If yes, what was your source of information?

1. Health worker (Doctor, Nurse, clinical officer)
2. Radio
3. Television
4. Newspapers
5. Others specify……………………………………….

21. Have you actually been involved in disclosure of HIV sero-positive to a child living with HIV?

1. Yes 2. No

22. Do you think it is important to disclose HIV sero-positive to a child living with HIV?

1. Yes (if yes go to question no.23)
2. No (if no go to question no. 24)

23. If yes give reasons___________________________

____

24. If no give reasons___________________________

____

25. Have you disclosed to your child his/her HIV sero-positive status?"

1. Yes
2. No

26. Have you discussed with the child why is he/she coming to the clinic?

1. Yes 2.No

27. Have you discussed about the soldiers of the body with your child

1. Yes 2. No

28. Have you told your child the name of the disease?

1. Yes (if yes go to Qn 29 to 36)
2. No (if no answer question number 31 then continue with qn no 37)

Determinants of fully HIV disclosure to HIV infected children

29. When was the child disclosed his/her HIV sero-positive status? (Mention the year)_____________

30. At what age was she/he disclosed sero-positive? ___________

31. What exactly did you tell the child about the illness which made him/her to attend this clinic?______________

32. At which class level did the child know his /her HIV sero-positive status?

1. Not in school
2. Kindergarten
3. Primary level
4. O' Level
5. Advanced secondary education
6. Others (Specify)________________________

33. What was the health of the child at the time you fully disclosed his/her HIV sero-positive status?

1. Very ill (admitted to the hospital)
2. Ill (Not admitted to the hospital)
3. Had no sickness
4. Others (Specify)______________

34. What prompted you to disclose the HIV sero-positive status of the child to him/her?

1. Physician advice/health care workers
2. Old enough
3. Family support
4. Low CD4 count of a child
5. Poor adherence to ARVs
6. Need to initiate ARVs
7. Others specify________________________

35. Who did the disclosure to the child?

1. Physician
2. Nurse
3. Counselor
4. Family friend
5. Other specify______________________

36. Where was disclosure done? ____________________________

1. Home

2. Hospital

3. School

4. Others (Specify)___________________________

37. What was the reason for not telling his/her HIV status?

1. Stigma
2. Fear of negative reaction from the child
3. It may disturb the child psychologically
4. May hurt child
5. Other reasons (specify))__________________________________

38. Do you have any plan of telling your child in future that he/she HIV infected?

1. Yes If yes go to question no.39   2. No

39. At what age are you planning to do that? _____________

40.Why do you think it is not possible for caregivers to inform the child his/her HIV status?__________

****************************THE END**********************************

CASE HISTORY GUIDE FOR CAREGIVERS WHO HAVE DISCLOSED HIV SERO-POSITIVE TO HIV INFECTED CHILDREN

Guide1; Disclosure of HIV sero-positive to HIV infected children has been mentioned a lot. Have you heard about HIV disclosure to HIV infected children? Tell me what do you know about HIV disclosure? Who informed you about it?

Guide2; what is your opinion concerning informing the child about the infection she/he has? Do you think it is appropriate for them to know what they are suffering from? Why? Could you give us the reasons?

Guide3; You told me before that your child knows his/her HIV. Its fine for your child to be informed about it, could you tell us what happened since you first came into contact with this child? (Processes) till he/she got to know that he/she is infected with HIV?

Probes; 1. When did you come into contact with the child (mention year).

Probe 2; The health of the child during this period of her/his life when you have been with him /her.

Probe 3; The time when the child got to know about her or his status.

Probe 4; How it started till the child understood he/she is infected?

Probe 5; Any assistance in disclosing to the child/who when and where?

Probe6; Any challenges in explaining to the child?

Probe 7; What happened thereafter (child reaction after finding out about the HIV_ status)?

KEY INFORMANT INTERVIEW GUIDE FOR HEALTH CARE PROVIDERS

Guide1;What are some of the issues you discuss with HIV infected children aged four to seventeen years/caregivers attending at Baylor's health facilities concerning HIV disclosure?

Guide 2; In your own opinion, do you think it's important to disclose to the children?

Guide3; Could you explain what processes do you go through to inform HIV infected children their HIV status from their first contact with Baylor clinic?

Probe1; Basing on the age of the child

Probe2; Health condition of the child

Probe 3; Family support

Guide4; Which approach would you recommend to be used in the processes of HIV disclosure to children? Why?

Guide 5; In your own opinion, how can HIV disclosure to HIV infected children improved so as to increase the number of children knowing their HIV status?

This is the end of interview thanks for your cooperation
